# Ethical and Social Aspects of Neurorobotics

**DOI:** 10.1007/s11948-020-00248-8

**Published:** 2020-07-22

**Authors:** Christine Aicardi, Simisola Akintoye, B. Tyr Fothergill, Manuel Guerrero, Gudrun Klinker, William Knight, Lars Klüver, Yannick Morel, Fabrice O. Morin, Bernd Carsten Stahl, Inga Ulnicane

**Affiliations:** 1 Centre for Computing and Social Responsibility, De Montfort University, The Gateway, Leicester, LE1 9BH UK; 2 King’s College London, London, UK; 3 Institute for Law, Justice and Society, De Montfort University, The Gateway, Leicester, LE1 9BH UK; 4 Centre for Research Ethics & Bioethics (CRB), Uppsala University, Uppsala, Sweden; 5 Department of Bioethics and Medical Humanities, University of Chile, Santiago, Chile; 6 Technical University Munich, Munich, Germany; 7 The Danish Board of Technology, Copenhagen, Denmark; 8 University of Maastricht, Maastricht, Netherlands

**Keywords:** Neurorobotics, Ethics, Responsible Research and Innovation, Human Brain Project

## Abstract

The interdisciplinary field of neurorobotics looks to neuroscience to overcome the limitations of modern robotics technology, to robotics to advance our understanding of the neural system’s inner workings, and to information technology to develop tools that support those complementary endeavours. The development of these technologies is still at an early stage, which makes them an ideal candidate for proactive and anticipatory ethical reflection. This article explains the current state of neurorobotics development within the Human Brain Project, originating from a close collaboration between the scientific and technical experts who drive neurorobotics innovation, and the humanities and social sciences scholars who provide contextualising and reflective capabilities. This article discusses some of the ethical issues which can reasonably be expected. On this basis, the article explores possible gaps identified within this collaborative, ethical reflection that calls for attention to ensure that the development of neurorobotics is ethically sound and socially acceptable and desirable.

## Introduction

Neurorobotics, the intersection of robotics and neuroscience, is an emerging area of research. It aims to find novel ways of controlling robots using neuro-inspired technologies, and provide embodiment for functional abstractions of (anatomical areas of) the brain, sometimes called brain models. As a cutting-edge field of scientific inquiry that builds on long-established traditions in its reference disciplines, yet aims to uncover entirely new ground, neurorobotics is an excellent area to explore how ethical and social concerns relate to scientific research and how science can engage with this type of concern.

Neurorobotics differs from other strands of robotics in that it attempts to bridge many areas of neuroscience and robotics to implement the neurobiological structures predicating animal and human behaviour in robots. This avenue of investigation has several scientific and technical aims. From the perspective of neuroscience, the ambition is to pursue a better understanding of the mechanisms underlying neural disorders.

The research question motivating the article arose from ongoing interaction between neuroroboticists and scholars working on Responsible Research and Innovation (RRI) within the Human Brain Project (HBP), with the explicit goal of proactively engaging with potential issues arising from developments in neurorobotics. This joint approach can be formulated as “which distinctive ethical and social issues may neurorobotics raise, and are mechanisms currently implemented sufficient to identify and address these?”

The article presents a range of issues, most of them also arising in other contexts, but coming to the fore in a different configuration in relation to neurorobotics. The article intends to lay the foundations for a systematic reflection on ethical and social implications of neurorobotics.

Hereafter, a discussion of ethics and traditional robotics is offered to provide relevant context. This is followed by an overview of neurorobotics, supported by a discussion of prototypical application areas for the technology. The article then presents analyses of several associated ethical and social concerns, followed by more detailed discussions of dual-use concerns, issues stemming from academia-industry collaboration, and data governance. The conclusion returns to the question of how RRI can help address these concerns.

## Ethics and Traditional Robotics Versus Neurorobotics

Humanity has long dreamed of autonomous robots which possess a broad set of skills, including perception, natural capacities for navigation and recognition of novel environments; attending to, aiding and working safely with others; goal-oriented behaviour, learning and decision making; a sense of self, and even consciousness. From an engineering perspective, desirable features of robotic systems include fault tolerance, low energy consumption, being lightweight and cheap, affording compliant mechanics and a compact design. In short, the engineer’s ambition consists of designing robots that display high levels of flexibility and adaptivity (Knoll 2016).

Strides in science and technology over the past few decades have made achievable what once remained a utopia. That is why some scholars have labelled the 21st century as “the age of the robots” (Brooks 2002). Nevertheless, such optimism is accompanied by concerns over how robotics and automation may change the way we work and, to an increasing extent, the way we live. If our time corresponds to “the age of the robots,” it is also true that we are living in a period that may be remembered as “the time in which the world woke up to imminent and likely impacts of developments in AI and robotics” (Prescott and Szollosy 2017, p. 121). Significant cultural, social, and economic changes have been attributed to the growing presence of robots in everyday life activities (IFR 2018). That is the case of the industrial robots, collaborative and social robots, distributed robotic systems, outdoor robots, health care, and surgical robots, military robots, educational robots, and entertainment robots (Veruggio 2006).

The corresponding social and ethical concerns have been summarised by the emergent field of robot ethics (or roboethics), a new applied ethics that is built in dialogue with the contributions of computer ethics, information ethics, bioethics, technoethics and neuroethics (IEEE 2004). Key issues discussed in roboethics include (Veruggio and Operto 2008; Ford 2015; Boden et al. 2011):

- the replacement of human beings by robots in the industrial and service sectors (a perspective intrinsically connected to some of the ambitions of neurorobotics);
- the potential misuse and dual-use of robots for warfare or terrorism;
- the anthropomorphisation of technological products, which attributes intentions, goals, emotions, and personalities to machines that could lead to social and psychological problems for the users;
- digital and socio-technological divides between generations, social groups and geographical regions;
- fair access to technological resources, and
- the environmental impact of technology.

The increasing integration of humans and artificial entities have raised safety, security, privacy, and reliability concerns, in direct relation to potential risks that autonomous systems present when they inhabit human environments. More specifically, the behavioural unpredictability, derived from the extension of autonomy and self-learning processes of robots, is a common matter of concern which has even been addressed by the European Union (Delvaux 2016).

Many of the considered issues are closely linked to the development of meaningful decisional autonomy for real robots, which is to a large extent caused by developments in Artificial Intelligence (AI), and its application to robotics. Ethical implications of AI are thus of direct relevance to this conversation. However, the purpose of this article is to look specifically at neurorobotics, a technology defined in the next section.

## Neurorobotics

Robotic technology suffers from clear limitations, both traditional and neurorobotics. The field of neurorobotics is located at the intersection of neuroscience and robotics. It is chiefly concerned with the functional connection of neural models with a physical or virtual embodiment. Robotics itself is a discipline revolving around physical interactions. What is usually referred to as robots are systems equipped with sensors, making available information relevant to the task pursued, and actuators used to act upon the environment. The essence of robotics lies in the enactment of apposite actions in light of sensor data. This connection, from perception to action, is where designers aim to impart a measure of intelligence to the system, with the robot’s quality often depending on the efficacy of the underlying algorithmics. Robotic technology is demonstrably capable of reliably performing a significant range of tasks. Robots are pervasive in the manufacturing industry (Nof 1999), they are used to support complex medical acts (Gomes 2011), and mobile robotic platforms have become indispensable tools in areas such as oceanography (Fiorelli et al. 2006) and inspection and maintenance (van Hoorn et al. 2018). The technology, however, suffers from clear limitations, both in the manner in which robots conceptualise their relationship to their environment and also in how they physically interact with it. Specifically, robotic systems typically have little capacity for abstraction or reasoning. As a result, robots lack meaningful decisional autonomy, contextual awareness, and struggle to adapt to evolving operating conditions. In addition, today’s robots still move very much like robots; that is, with movements that are stiff, mechanical, and entail functional limitations.

In their search for ways to overcome these challenges, roboticists came to consider different paradigms. In particular, the idea of turning to neuroscience to improve robotics became widespread in European bio-inspired robotic projects of the early 2000s (Dario et al. 2005), with promises of greater agility and improved environmental perception. However, while bio-inspiration did allow for the development of novel actuation and sensing modalities (Pfeifer et al. 2007), robots have seemingly been unable to capitalise on this potential. Part of the issue can be tracked to the algorithms processing sensor measures and assigning actuator commands, which have historically been developed using results from the dynamics and control theory literature (Khalil 1996; Isidori 2013). Natural agents, however, have demonstrated expert use of these same (perception and locomotion) modalities by relying on neural systems to process afferent information and generate motor commands. Neurorobotics thus proposes to emulate nature in this particular respect, considering the use of neural models to develop algorithmic solutions for robotic applications. It explores what neuroscience may contribute to robotics at all three levels of Marr’s classification: computational, algorithmic, and implementation-related (Marr and Poggio 1976).

The approach taken within the HBP provides opportunities and tools for neuroscientists to explore the functional properties of brain models of interest in an embodied setting, either within simulated agents in physically realistic (although abstracted and approximated) numerical environments, or directly in physical robots. This embodiment provides a unique opportunity to observe and understand how neuronal dynamics interact, at multiple time scales, to produce behavioural responses when exposed to realistic inputs, such as properly correlated sensory streams. For neuroscience specifically, this aspect of embodiment brings about a significant dimension: loop-closure (in the dynamical system theory sense). Neurorobotics is specifically interested in reflecting the manner in which the action-perception loop is closed; the agent acts on the environment, the environment reacts, the agent perceives the reaction. The implication being that the physical incarnation, provided to the studied neural model, can more naturally interact with its environment. The prospect of studying neural systems (in particular those involved with sensorimotor functions) in closed-loop is a particularly attractive one, giving rise to the notion of closed-loop neuroscience (Potter et al. 2014).

The information and communication technology (ICT) tools developed by the HBP were built specifically around these concepts of embodiment and closed-loop neuroscience. They may as such be envisioned to support the emergence of a new class of robots and, in the longer term, of neuro-technology and neuro-engineering approaches for prosthetic devices (Wagner et al. 2018). The particular nature of these tools, however, entails specific ethical issues, insofar as they support the use of neuroscientific knowledge as a means to an end, and make this process accessible (through open platforms and brain atlases), customisable (open source software), and amenable to automation (e.g. standardisation of data formats, use of knowledge graphs, etc.). Accordingly, it stands to reason that HBP-enabled neurorobotics should not limit itself to considering the ethical issues associated with potentially providing artificial devices with advanced or even human-like cognitive structures. It should also pay special heed to the possibility of human intentions and biases injecting themselves into this process.

The limited maturity of neurorobotic technology makes it difficult to assess which particular (anticipated) ethical issues may in time become prevalent. In addition, the manner in which such concerns relate to existing ethical issues commonly affiliated with traditional robotics is not entirely transparent. To make this discussion more concrete, the discussion hereafter focuses on a number of application areas, the narrower focus lending itself to a more detailed ethical analysis.

## Possible Applications of Neurorobotics: Potential Opportunities and Ethical Questions

In the following, the discussion directly addresses specific application areas of special relevance for neurorobotics, concerned with technical (industrial robotics and automation), neuroscientific (medicine), and societal aspects (education).

Industrial robots and humans have complementary skills, allowing robots and humans to work together on the same task thus constitutes an interesting proposition. However, this premise finds itself, in practice, hampered by several limitations. Safety requirements limit the movement speed of robotic arms in proximity to humans (Zanchettin et al. 2016), which reduces productivity and renders such collaboration impractical. If traditional robotic technology has thus far failed to realise this potential, ongoing developments in neurorobotics may give rise to practical solutions. In particular, neurorobotic results on multimodal perception and contextual awareness (emulation of brain structure and functions in this respect), could provide solutions for robust estimation of a human worker’s space occupancy (within the workspace of the robot), with quantified performance guarantees, thereby enabling relaxation of speed limitations on the robot without jeopardizing the safety of the worker. The short-term benefits are clear; combining robot and human skills can improve productivity (e.g. assembly in the automotive industry). In addition, allowing workers to more frequently rely on robots for manipulation of heavy loads will improve working conditions, have a positive impact on health, and alleviate the corresponding socio-economic burden. In the longer-term, the improved productivity can improve the competitiveness of the industrial sector, positively impact the economy, and contribute to the re-domiciliation of industrial production capacities in developed countries.

A fundamentally different application area of neurorobots is in medical research. A tool such as the HBP’s Neurorobotics Platform can conceptually be used to model and better understand the source of a given pathology in terms of malfunctioning neuronal circuitry. Movement disorders such as Parkinson’s, in particular, will benefit from embodied simulations that include all brain areas potentially affected by the disease (e.g. basal ganglia, but also motor cortex, cerebellum, etc.). Embodiment provides the tools to directly connect motor symptoms to neuronal function, in a reproducible and testable manner, while at the same time accounting for a robust range of confounding parameters, such as proprioceptive signals, or mechanical compliance of the human body. The potential ethical and scientific benefits of this approach are far-ranging. From a neuroscientific perspective, such work can be used to test the merit of neuroscientific models and hypotheses. This can inform medical interventions and treatments, for example of spinal cord injuries (Wagner et al. 2018). A possible ethical advantage could be that reliance on animal models might be reduced, which would have animal welfare benefits and help promote the 3Rs—Replacement, Reduction, and Refinement in keeping with Directive 2010/63/EU of the European Parliament and of the Council of 22 September 2010 on the protection of animals used for scientific purposes.

A third example of neurorobotics applications is adaptive learning schemes for ubiquitous and immersive human–computer interaction. Neurorobotic learning schemes are being investigated as enabling technologies to train robots and other computer systems that serve as interfaces for humans in real environments (virtual and augmented reality, as well as mobile, wearable, tangible and ubiquitous computing with multi-modal feedback). To these ends, robots and computer systems function both as interfaces and as real objects to be controlled by other user interfaces.

## Social and Ethical Concerns of Neurorobotics

Social and ethical concerns raised by neurorobotics can cover the same issues as those raised by traditional robots and more generally by other novel technologies that affect people’s everyday activities. However, neurorobotics can also generate novel concerns. The novelty originates from bio-inspired robotics and biomechatronics. That is the case, for instance, of the bi-directional translation that characterises neurorobotics, which links brain research to information technology, designing computational structures for robots inspired by the study of the nervous systems of humans and animals, and using the study of bio-inspired robots to improve the understanding of the human brain. As a result, the ethical and social concerns raised by the traditional robotics can acquire a new meaning and philosophical depth. Neurorobotics aims to endow machines with greater awareness, capacity for abstraction, reasoning; in short, with a greater degree of intelligence - a quality we have come to associate with humanity. It does so by attempting to emulate the mechanisms that predicate intelligence in humans, for which the nervous system provides a physical manifestation. What does this say about the relative condition of man and machine? Do they exist along a common spectrum? Is this a rational pursuit? Alternatively, is the notion that we may analyse, understand and reproduce what makes us human rooted in something other than reason?

In addition, as neurorobotics aims to improve the capabilities of existing robotic systems and, as a consequence, extend the use made of robotics and automation technology, some of the existing concerns can be exacerbated. This is particularly the case for those application areas that stand to directly benefit from advances brought about by neurorobotics, such as those discussed earlier.

### Responsible Research and Innovation in the HBP

This section provides a brief introduction to the way in which broader ethical and social questions are being addressed in the HBP. This overview helps to contextualise this article, which arose from such work in the project. The HBP brings together a large number of people from a broad range of disciplines to work on several potentially contentious and sensitive topics, ranging from animal experimentation to the possibility of conscious machines (Rose 2014; Lim 2014). It, therefore, incorporated a Society and Ethics sub-project from its inception. This Ethics and Society Programme adopts the principles and practices of Responsible Research and Innovation (RRI). RRI is a key concept in research and innovation governance, which in the European framework programme Horizon 2020, concentrated on ethics, governance, open access, science education, public engagement and gender equality (European Commission 2013). It is based on the attempt to render research and innovation acceptable, socially desirable and sustainable (von Schomberg 2011). The UK Engineering and Physical Sciences Research Council has established the so-called AREA framework for RRI, which is an acronym for anticipation, reflection, engagement, and action. This AREA framework is implemented in the HBP (Stahl et al. 2019).

In practice, the RRI programme of the HBP is implemented by four work packages that focus on technology foresight (Aicardi et al. 2018), philosophical and neuroethical reflection (Salles et al. 2019), public engagement, and ethics support (Stahl et al. 2016). Ethics Support uses an approach of ethics dialogues to implement RRI (Stahl et al. 2019). As part of the ethics-related work of the RRI programme, the scholars in the programme collaborate with colleagues from the scientific and technical disciplines to identify and address ethical challenges. This article is the result of an engagement that brought together researchers in neurorobotics with members of the RRI group.

The following discussion draws on the work of the RRI group with a particular emphasis on several of the topics that were identified previously. These sections focus on the following issues that are all well-researched and discussed, but which take on new urgency in neurorobotics, namely dual use, collaboration between academia and industry, and data governance.

### Dual Use

As the discussion above suggests, misuse or harmful use is one of the ethical concerns affecting neurorobotics in particular and neurotechnology in general (see also Ienca et al. 2018). General dual-use and misuse issues in neurorobotics include potential future military applications of mobile robotics, amongst others. More specifically within the HBP, concerns have been raised about potential dual-use and misuse of neurorobotics platform tools. The HBP Ethics and Society division’s approach to address these issues is set out in its “Opinion on ‘Responsible Dual Use’: Political, Security, Intelligence and Military Research of Concern in Neuroscience and Neurotechnology” (Ethics & Society 2018). This approach to dual use issues is broader than the one set out in the EU Horizon 2020 policies (European Commission 2019). In line with the overall principles and practices of RRI in the HBP described above, responsible dual-use approach implements the AREA framework to facilitate anticipation, reflection, engagement and action and is implemented through ethics dialogues (Ulnicane 2020). Moreover, it draws on the concept of ‘dual-use research of concern’ (DURC) that the US government applies to prevent the malicious application of life science research.

As with all research projects funded by the EU Horizon 2020, the HBP has to have an exclusive focus on civil applications. According to EU policy, “dual-use items are normally used for civilian purposes but may have military applications, or may contribute to the proliferation of weapons of mass destruction” (European Commission 2019, p. 33). The Horizon 2020 policy does not rule out dual-use research, i.e. ‘the development of generic technologies, products or knowledge that may meet the needs of both civil and military end-users (known as ‘dual-use’ goods or technologies), provided that the research itself has a clear focus on civil applications’ (European Commission 2019, p. 35).

The work of the HBP Ethics and Society division (Ethics & Society 2018) has identified several limitations and ambiguities in the EU approach. First, it is based on a civil-military use dichotomy, which fits an outdated, historical understanding of the dual use concept (see e.g. Molas-Gallart 1997) that over time has been expanded to refer to research and technology that can be used for both beneficial and harmful purposes in a broader sense (e.g. Ienca et al. 2018; Oltmann 2015). To highlight this broader range of potentially beneficial and harmful uses of neuroscience, HBP partners have explored other types of applications, for example, political, security and intelligence (Mahfoud et al. 2018). Second, the EU definitions of dual-use quoted above (based on the EU export control regulations), focus on items, goods, and technologies. However, in science project such as the HBP, a lot of research and development is still at an early stage (which is also the case for neurorobotics) i.e. well before potential uses have crystallised and the export of goods, items and technologies can be considered. The RRI approach pursued in the HBP allows us to facilitate early anticipation and reflection on potential uses, develop ways to support responsible use and avoid misuse.

Thus, the responsible dual-use approach developed in the HBP has a strong focus on involving scientists and engineers early on in anticipation and reflection on potential uses of their research as well as on engaging stakeholders and policy-makers. Responsibility in this context refers to

processes and practices within research and development systems, and the extent to which they encourage or constrain capacity of all those involved in the management and operation of research to reflect upon, anticipate and consider the potential social and ethical implications of their research, to encourage open discussion of these, with a view to ensuring that their research and development does indeed contribute to the health and well-being of citizens, and to peace and security. (Ethics & Society 2018, p. 9)

Moreover, according to the Ethics and Society division’s Opinion, the introduction of RRI can enable clarification of what might count as Dual Use Research of Concern.

### Academic Research–Industry Partnerships

As outlined earlier, neurorobotics is emerging at the confluence of neuroscience and robotics, with the potential for a wide array of industrial, medical and healthcare applications. It, therefore, attracts interest from prospective partnerships between public research and industry. In the European Union and European countries, cooperation and transfer of knowledge and technology between public research and industry are seen as key to delivering research and innovation for economic growth and societal impact. For all their expected benefits, these partnerships face recurring barriers that can lead to problematic consequences, and that raise significant ethical and social concerns which reflect broad issues common to research areas with such high application potential as neurorobotics.

Orientation-related barriers to relations between academic research and industry are well-identified (divergences in goals, in time horizons, in working practices, in expectations, in incentives), yet transaction-related barriers are also important and appear much more difficult to mitigate. The latter particularly relate to intellectual property (IP) management and administrative procedures (e.g. overselling of research and unrealistic expectations by Technology Transfer Offices, financial or regulatory conflicts over patents and other IP rights) (Bruneel et al. 2010). Rather counter-intuitively, policies forcefully pushing universities to develop research commercialization may actually have increased transaction-related barriers (Bruneel et al. 2010; Hall et al. 2014). Questions surrounding IP may be particularly pertinent for neurorobotics—for instance, whether it should be possible to patent a control structure generic to the human brain or even specific to one human brain, which ties into essential ethical discussions such as gene patenting. These questions, in particular, the potential increase of transaction-related barriers induced by forceful commercialisation strategies, are moreover very pertinent for the HBP itself, and thus for neurorobotics as a research programme requiring the kind of large interdisciplinary collaborative organisation exemplified by the HBP.

Bruneel et al. (2010) identify the following factors as beneficial to cooperation between academic research and industry: inter-organisational trust, the experience of collaboration, and the breadth of interaction channels. An important way to encourage these is to think about partnerships in much broader terms than strict technology transfer: collaborative research (e.g., informal interactions, institutional agreements, research consultancy), knowledge transfer (e.g., researchers and recent graduates hiring, cooperative education, secondments), and research support (e.g., endowment, shared facilities) (Bruneel et al. 2010; Mascarenhas et al. 2018; Perkmann et al. 2013).

Finally, academic research collaborating with industry raises significant ethical and social concerns in itself. Research independence and integrity of research are a high priority, as is avoiding conflicts of interest, professional misconduct, and double binds (Evans and Packham 2003). Then, there is a concern regarding the privatisation of common goods: valuable research outcomes may only benefit the interests of a few, while support for the research process as a whole, including its inevitable failures, is mutualised across European citizens through their taxes (Rose et al. 2015). Another issue is the little-investigated impact that academic researchers’ external engagements can have on their academic missions of research and teaching (Perkmann et al. 2013). Neurorobotics’ overarching goal of bridging between neuroscience and robotics carries with it the real risk that neurorobotics may become pulled into different directions by the interests of neuroscientific research and robotics applications. Compounded by the disproportionate capacity for investment by big industrial players compared to public research funding bodies, this raises the issue that foreseen applicative benefits may start weighing on the research agenda of neurorobotics to the detriment of neuroscience. The pull of promissory innovation and socio-economic benefits could even lead neuroscientific research to privilege its translation potential for neurorobotics applications over other objectives.

### Data Governance

A final set of concerns to discuss relates to data governance. Data governance is essential to responsible innovation in the increasingly global context of neuroscience and ICT research, particularly considering the importance of big data analytics for the general advancement of neuroscience. Neurorobotics is no exception to this, and the innate complexity of these endeavours presents some specific challenges. There are potentially competing calls for openness and transparency (Salerno et al. 2017), and parallel concerns regarding data security (Siddiqa et al. 2016), privacy and data protection. In addition to negotiating this tension, data governance processes must attempt to prepare for unpredictable futures and address issues of intellectual property, authorship, and contributor credit since ownership of data is not always straightforward and may be shared or contested. Neurorobotics raises specific data governance challenges. In pursuing the unique set of approaches characteristic of neurorobotics, linking simulated “brains” with physical sensors; working toward novel control technologies; and creating experimental design and simulation, a complex and overlapping data lifecycle is generated. This is significant in terms of developing appropriate data governance strategies because these are contextually contingent upon data lifecycle phases and distinct ethical issues arise at different stages (Fothergill et al. 2019). In the longer term, this could present possibilities for unforeseen vulnerabilities or security lapses and may increase the risk of a loss of data fidelity or misallocation of metadata, which could result in a data breach or threaten replicability. Such potential concerns, coupled with the disciplinary diversity within neurorobotics, necessitate a data governance approach which is fully grounded in a theoretical awareness of the need for integrated ethical frameworks within innovation processes.

## Conclusion

This article addresses the question of which social and ethical issues are raised by neurorobotics. By clarifying the concept and specific application areas, the article provides insights into the potential capabilities of neurorobotics, and on the specific impact, this emerging technology may have on our everyday lives. A critical insight arising from the analysis presented is that ethical issues related to neurorobotics are neither radically novel nor surprising. The various ethical considerations discussed in this article are generally rather well established in the literature. Nevertheless, it is apparent that neurorobotics may raise or exacerbate concerns, such as those related to worker safety, systems reliability, or the introduction of unconscious biases.

This is not to say, however, one should not remain mindful of the ethics of neurorobotics. If the work presented here tends to show that, at this early stage of development of neurorobotics, immediate intervention is not necessarily warranted, some of the perspectives drawn seem to deserve our attention. It remains difficult to anticipate how and where neurorobotics will be used, and the extent to which novel application areas may give rise to new ethical challenges.

An important conclusion to be drawn from this description is that ethical reflection of neurorobotics needs to be an ongoing activity that has to be driven and informed by scientific and technical expertise. The article should be read in this spirit, as an important first step on a journey of establishing a collaboration centred on neurorobotics and reflexive research from the social sciences and humanities. The aim of this journey is a clear understanding of what responsible neurorobotics would look like. The concept and practices of RRI are well-suited to inform this journey. Processes of RRI and ethics dialogues that are in place in the HBP offer a robust starting point. They provide the basis for the type of interdisciplinary collaboration that allows the exchange of views and ideas that have resulted in this article. They also allow for bringing in external stakeholder voices and other types of views and concerns.

The next and more challenging steps will be to develop structures that help to address these issues and that can offer practical guidance. The framework of RRI provides a good basis for such structures; the principles of anticipation, reflection, engagement, and action being at the heart of practical solutions. Such principles also offer a reliable foundation to support the implementation of the higher-level and policy-oriented recommendations suggested above.

That this article does not answer all questions raised and does not articulate the detail of practical next steps should not be seen as a limitation. Rather, it is an illustration of the fact that ethical and social concerns should continuously remain a meaningful dimension of our thought process, as we undertake research and innovation activities.
